# Hierarchically coupled multiscale structure–property correlations in electrodeposited Zn–TiO_2_–graphene oxide nanocomposite coatings

**DOI:** 10.1039/d6ra04122a

**Published:** 2026-08-03

**Authors:** Durgesh Phogat, Megha Goyal, Wannaporn Senachamnong, Ankur Srivastava, Shikha Awasthi

**Affiliations:** a Department of Chemistry, Manipal University Jaipur Rajasthan 303007 India; b Department of Industrial Chemistry, King Mongkut's University of Technology North Bangkok 10800 Thailand; c Department of Mechanical Engineering, Manipal University Jaipur Rajasthan 303007 India ankur.srivastava@jaipur.manipal.edu; d Jagran School of Pharmacy, Jagran Lakecity University Bhopal – 462044 Madhya Pradesh India awas.shikha2212@gmail.com

## Abstract

Electrochemically deposited zinc (Zn)-based composite coatings have multifunctional applications in corrosion prevention, mechanical strengthening, photocatalysis, biomedical implants, energy storage, and optoelectronics. In the present study, titanium dioxide (TiO_2_) nanoparticles and graphene oxide (GrO) sheets were co-electrodeposited within a Zn matrix on copper substrates to produce pristine Zn, Zn–TiO_2_, Zn–GrO, and Zn–TiO_2_–GrO coatings. The morphology, composition, crystal structure, wettability, surface roughness, and mechanical properties of the coatings were systematically investigated. The Zn–TiO_2_–GrO hybrid coating exhibited the best overall performance, with improved hydrophilicity (44.2°), lowest surface roughness (0.157 ± 0.06 µm), refined crystallite size (18 ± 2 nm), and the highest hardness values (29 HV). Chen model calculations indicated enhanced elastic modulus values for the composite coatings (up to 12.70 GPa) compared with pristine Zn (3.25 GPa). The improved performance of the hybrid coating is attributed to the synergistic effect of TiO_2_ nanoparticles and graphene oxide, which promotes grain refinement, improves interfacial bonding, and enhances load transfer within the Zn matrix. Thus, this study reveals that the Zn–TiO_2_–GrO composite coating provides an effective strategy for multifunctional applications with enhanced surface and mechanical properties.

## Abstract 2

Zn–TiO_2_–GrO composite coatings exhibited refined grains, enhanced interfacial bonding, improved surface morphology, higher mechanical strength and greater hydrophilicity than bare Zn.

## Graphical abstract

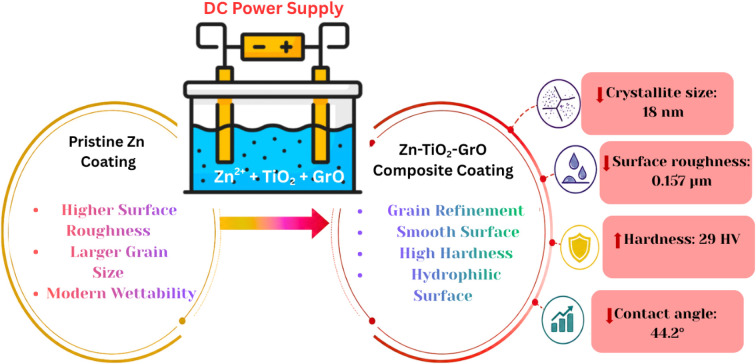

## 1. Introduction

Zinc (Zn) is emerging as a scientific and industrial material due to its wide functional and technological adaptability, with an inherent hexagonal crystal lattice and strong piezoelectric behaviour, which makes it highly efficient for the integration of multifunctional and MEMS-based appliances.^1^ Zn has received significant interest in the scientific and industrial fields due to its broad functional versatility, economic sustainability, and eco-friendliness.^2^ The inherent hexagonal wurtzite crystal lattice is conducive to Zn with strong piezoelectric characteristics, making it susceptible to multifunctional and MEMS-based device integration.^3^ ZnO is a Group II–VI semiconductor that exhibits optical transparency, high electrical conductivity, strong room-temperature photoluminescence, and excellent chemical stability, making it attractive for photocatalytic, photovoltaic, UV-shielding, and gas-sensing applications.^4^ Although these intrinsic advantages, pristine Zn coatings show moderate mechanical strength, low hardness, and tribo-corrosive degradation at low temperatures or physicochemical environments. These deficiencies compromise their strength and corrosion resistance in terms of durability, particularly in acidic or alkaline environments.^5^

To eliminate these disadvantages, microstructural refinement, defect modulation, and composite reinforcement methods are widely discussed to maximise their functional performance, and durable nanoparticles have been especially effective in enhancing surface performance.^6^ Klekotka *et al.*^7^ electrodeposited Zn-metal oxide nanocomposites, which exhibited better hardness (124 HV) and lower coefficient of friction (0.68) compared to unmodified Zn due to the load-bearing capacity and interfacial stress dissipation, which enable hard nanoparticle reinforcement. Surface-engineering methods, such as oxy-fuel spraying at high velocity, micro-arc oxidation, plasma alloying, laser cladding, and electrochemical deposition, are cost-effective and scalable methods, providing precise measurement of coating thickness, topography, and interfacial cohesion.^8^ Consequently, the integration of reinforcing nanophases into Zn matrices has become a critical design paradigm for developing coatings with tailored mechanical strength, wear, and corrosion resistance suited to advanced engineering and biomedical applications.^9^

With continual advancements in the Zn-based composite design, graphene oxide (GrO) and titanium dioxide (TiO_2_) have been of particular interest as additives that can be used to augment the multifunctional response of Zn coatings. Zn-based composite coatings have inherently pronounced thermal stability and inherent corrosion resistance. These hybrid systems are also promising in electrochemical energy storage (batteries and supercapacitors), biomedical applications such as drug delivery, osseointegration, and tissue regeneration, and new optoelectronic and sensing platforms. Altaf *et al.*^10^ synthesised a Zn–GrO nanocomposite coating *via* electrophoretic deposition (EPD), and nanoindentation testing revealed that the hardness and elastic modulus (14.8 GPa and 190.9 GPa, respectively) of the coating had significantly improved in comparison to the pristine Zn coating (7.4 GPa and 132.2 GPa, respectively). The synergistic toughening mechanisms, *i.e.*, crack bridging, grain bridging, and crack deflection caused by the GrO nanofillers, were attributed to the high mechanical strength and interfacial rigidity of the Zn–GrO composite coating that effectively hindered crack propagation and redistributed localised stresses under mechanical loading. The degree of nanoparticle incorporation during electrodeposition largely depends on the dispersion stability of the particles in the plating bath. To minimize particle agglomeration and ensure uniform particle distribution, surfactants are commonly employed. Among the various surfactant classes, anionic surfactants such as sodium dodecyl sulfate (SDS) are particularly effective because they provide electrostatic stabilization to suspended nanoparticles, thereby improving their dispersion and facilitating their incorporation into the growing metal matrix. Consequently, the use of SDS promotes the formation of more homogeneous Zn-based nanocomposite coatings with improved microstructural uniformity.

Ceramic structures such as titanium dioxide (TiO_2_) exhibit broad multifunctionality, including optical brightness enhancement, opacity regulation in paper, whitening in food and cosmetic formulations, pharmaceutical excipients, and coating agents, as well as advanced applications such as photocatalysis-driven surface activity and self-cleaning coating functionalities.^11^ Praveen *et al.*^12^ fabricated Zn–TiO_2_ composite coatings *via* electrophoretic deposition (EPD), systematically varying the applied potential (10–40 V) over 0.5–8 min. Reduced crack density, particularly at 20 V, indicated improved film uniformity and structural coherence, while potentiodynamic polarisation and electrochemical impedance spectroscopy (EIS) confirmed enhanced corrosion resistance in simulated body fluid (SBF). Additionally, osteoblastic cells cultured on the hybrid coatings demonstrated excellent proliferation and morphology, reflecting high cytocompatibility and biofunctional performance. The strategic incorporation of such multifunctional reinforcements, therefore, strengthens mechanical resilience, improves structural integrity, and enhances biofunctional efficacy through synergistic interfacial interactions and increased surface bioactivity.^13^

This study focuses on creating new composite coatings (Zn, Zn–TiO_2_, Zn–GrO and Zn–TiO_2_–GrO) on copper substrates to enhance multifunctional behaviour. Numerous studies have reported various characteristics of Zn-based metal matrix composite coatings fabricated under different processing conditions; however, a consistent comparison has not been conducted. The electrodeposition of Zn composite coatings with the reinforcement of GrO and TiO_2_ nanoparticles is relatively untouched. The ability of such hybrid coatings to perform depends to a large extent on the homogeneous dispersion of the reinforcement phases and strong interactions between the reinforcement phases and the Zn matrix that affect the mechanical and functional properties.

## 2. Experimental studies

### 2.1 Materials

Zinc sulfate (ZnSO_4_·7H_2_O, 99% purity), sodium chloride (NaCl, 99% purity), and sodium lauryl sulfate (SLS, 99% purity) were employed as zinc precursors and electrolyte constituents in the electrodeposition process. Titanium dioxide (TiO_2_) and graphene oxide (GrO) nanoparticles, utilised as reinforcing nanophases, were procured from Sarthak Sales, Jaipur, India. Analytical-grade reagents were employed throughout the study without any additional purification.

### 2.2 Preparation of substrate and electrodeposition of Zn composite coatings

The electrodeposition of pristine Zn and Zn-based hybrid nanocomposite coatings was conducted in a two-electrode electrochemical cell under direct current (DC) regulation. Copper substrates were chemically treated in 10% HCl before deposition, cleaned off surface oxides, and polished by successive mechanical operations with emery papers of finer and finer grit until a mirror finish was obtained. The specimens were then ultrasonically cleaned with deionised water to remove any residual impurities. The electrolyte bath comprised ZnSO_4_·7H_2_O (150 g L^−1^), Na_2_SO_4_ (150 g L^−1^), NaCl (30 g L^−1^), and sodium dodecyl sulfate (SDS, 0.05 g L^−1^) as a surfactant, as shown in Table 1. Based on its ability to provide electrostatic stabilization and reduce particle agglomeration, sodium dodecyl sulfate (SDS) was selected as the anionic surfactant for the present study. The solution was magnetically stirred at 200 rpm and kept at an acidic pH of 2–3. A polished copper plate was used as the cathode (working electrode), and a platinum foil was used as the anode (counter electrode). To develop distinct coating systems, TiO_2_ and GrO nanopowders were incorporated into the electrolyte (0.5 g L^−1^ each), yielding four single baths: pristine Zn, Zn–TiO_2_, Zn–GrO, and Zn–TiO_2_–GrO. Electrodeposition was carried out for 30 min at a constant cell potential of 3.0 V and a current density of 3.0 mA cm^−2^ under ambient conditions.^14^

**Table 1:** Nomenclature and chemical composition of the fabricated Zn-based coatings

Description of samples	Nomenclature	Chemical composition
Zn coating	Zn	Zn base bath-ZnSO_4_: 150 g L^−1^
		NaCl: 30 g L^−1^
		SDS: 0.05 g L^−1^
Zn coating with TiO_2_	Zn–TiO_2_	Zn base bath + TiO_2_-0.5 g L^−1^
Zn coating with GrO	Zn–GrO	Zn base bath + GrO-0.5 g L^−1^
Zn coating with TiO_2_ and GrO	Zn–TiO_2_–GrO	Zn base bath + TiO_2_ + GrO

### 2.3 Physicochemical characterisation of Zn composite coatings

The surface morphology and elemental distribution of the electrodeposited coatings were investigated *via* field emission scanning electron microscopy (FE-SEM, Ultra 55, Carl Zeiss, Germany) integrated with energy-dispersive X-ray spectroscopy (EDX; EDAX ECON-4, EDAX, Mahwah, NJ, USA). The FE-SEM imaging parameters were standardised using a gold-on-carbon calibration grid (Ted Pella Inc., USA) to ensure dimensional and magnification accuracy. The crystalline phase constitution and lattice texture were delineated using an X-ray diffractometer (XRD-6000, Shimadzu, Kyoto, Japan) equipped with Cu Kα radiation (*λ* = 1.5406 Å) operating under the Bragg–Brentano geometry. Instrumental calibration was verified against a silicon standard (NIST SRM 640d) to maintain 2*θ* accuracy. Diffraction data were acquired within the 2*θ* range of 10–80° with a step interval of 0.02° and a dwell time of 1 s per step at 40 kV and 30 mA. The average crystallite size (*t*) was calculated employing the Scherrer relation:1
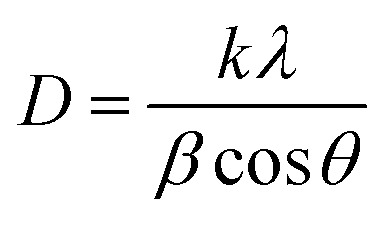
where *k* = 0.9 (shape factor), *λ* = 1.5406 Å, *β* = FWHM of the peak, and *θ* = Bragg angle. In addition, Fourier Transform Infrared Spectroscopy (FTIR) analysis was conducted with a constant aperture size of 100 µm diameter and an Hg–Cd–Te (mercury–cadmium–telluride) sensor, were identified using a Bruker Alpha FTIR spectrophotometer within the 4000–500 cm^−1^ wavenumber range.

### 2.4 Surface roughness and contact angle

To gain complementary micro- and nanoscale information, both a contact-type surface profilometer (Mitutoyo Surftest SJ-210) and atomic force microscopy (AFM) were used to characterise the surface roughness of the fabricated specimens. The profilometer is based on the principle of measuring the height variations of the surface by means of a diamond-tipped stylus that scans the surface mechanically, and the measured analogue signal is converted to digital roughness parameters (*R*_a_, *R*_q_). AFM can obtain the surface topography in three dimensions at the nanoscale, allowing for detailed analysis of surface morphology and height distribution. These techniques provide a complete and accurate characterization of the surface roughness and topography of the coatings. Contact angle measurement is a fundamental and widely employed technique for explaining surface physicochemical properties over a wide range of technological domains, such as specialised wetting systems, ink-jet deposition, and spray-cooling methodologies. The contact angle, denoted as *θ*_s_, is a macroscopic expression of the complex interfacial processes between a liquid phase and a solid substrate. This parameter provides critical information on surface energy, chemical composition, and morphological capillary phenomena, features, and capillary phenomena on a micro- and nanometric scale. In the current investigation, an optical contact angle (OCA) goniometer (Data Physics OCA 15 EC model) was used to determine the contact angle measurements, which were made by capturing the two values of the left and right contact angles of the sessile drop, and the average of these values was then used to analyse quantitatively. The wettability of the coatings was quantitatively measured through measurements of the contact angle of water in stasis, using a 5 µL sessile droplet. The contact angle is the angle established between a liquid droplet and the underlying surface at their point of contact^15^

### 2.5 Experimental and theoretical mechanical strength of the coatings

Hardness is the resistance of a material to localised surface deformation, including scratching or indentation, and is a measure of wear and abrasion resistance. It is the resistance of a material to plastic deformation or penetration by an indenter. Hardness and tensile strength are two different mechanical responses, but they are roughly proportional in copper. The hardness was measured in this study by the Rockwell and Vickers methods. The Rockwell hardness test is based on measuring the difference in indentation depth between a minor preload and a major applied load. The preload is a reference position, followed by the major load to obtain maximum penetration. The depth of the indentation left after unloading is recorded and is called the Rockwell hardness number (HRC). Rockwell hardness measurements were performed under a load of 60 kgf. In addition, Vickers microhardness testing was conducted using a diamond pyramidal indenter with an applied load of 100 g and a dwell time of 15 s. The hardness value (HV) was calculated from the applied load and the average diagonal length of the residual indentation. Five indentations were made on each sample, and the average hardness value was reported to ensure accuracy and reproducibility.^16^ For a more rigorous assessment of reinforcement integration within the matrix, the elastic modulus of the Zn-based composite coatings (Zn–TiO_2_, Zn–TiO_2_–GrO, and Zn–GrO) was evaluated using various micromechanical models, including the rule of mixtures (ROM), the combined Voigt–Reuss (V–R) and Hill model for upper and lower bounds.^17^ The ROM yields markedly distinct upper and lower bounds, considering solely the volume fractions of reinforcement and matrix (*V*_r_ and *V*_m_). The V–R model incorporates the volume fractions of reinforcement alongside the shear moduli of reinforcement and matrix (*G*_r_ and *G*_m_), forecasting the elastic modulus *via* upper and lower bounds.

### 2.6 Statistical analysis

The experiments were conducted in triplicate (*n* = 3), and the average value was calculated along with the standard deviation for all testing related to Zn-based composite coatings.

## 3. Results and discussion

The physicochemical, morphological, wettability, and mechanical characteristics of the electrodeposited Zn-based composite coatings were systematically investigated to evaluate the influence of TiO_2_ and graphene oxide reinforcements. The results are discussed sequentially, beginning with microstructural and compositional analyses, followed by phase identification, surface wettability, roughness, and mechanical performance. The combined interpretation of these results provides insight into the synergistic effects of TiO_2_ and graphene oxide incorporation on the structural integrity and functional behaviour of the Zn-based composite coatings.

### 3.1 Surface morphology, phase structure, and coating thickness

**Fig. 1:**
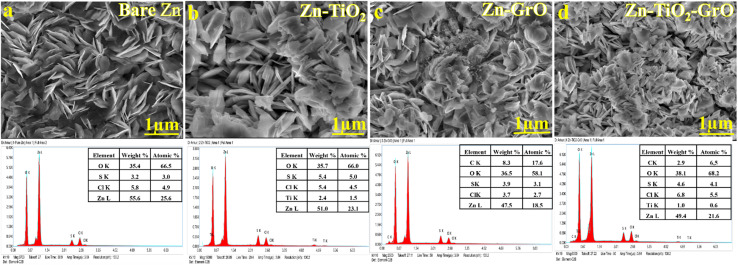
The surface morphology of the Zn composite coatings is presented in (a) Pristine Zn, (b) Zn–TiO_2_, (c) Zn–GrO, and (d) Zn–TiO_2_–GrO, respectively.

Cross-sectional micrographs of the electrochemically deposited Zn, Zn–TiO_2_, Zn–GrO, and Zn–TiO_2_–GrO coatings are depicted in Fig. 1a–d, exhibiting thicknesses around 64–85 µm (Table 2). The electrochemically deposited Zn, Zn–TiO_2_, Zn–GrO, and Zn–TiO_2_–GrO coatings exhibit dense, adherent, uniform, and continuous microstructures, consistent with optimised electrodeposition parameters for enhanced mechanical integrity. The top surface morphology of the pristine Zn coating (Fig. 1a) reveals a featureless topography, whereas TiO_2_ particles are homogeneously dispersed within the Zn matrix of the Zn–TiO_2_ coating, devoid of agglomerates (Fig. 1b). Flower-like morphological patterns are evident in the Zn–GrO and Zn–TiO_2_–GrO composites (Fig. 1c and d), a feature widely documented in electrodeposited Zn-based nanocomposites and attributed to preferential nucleation and growth kinetics.

**Table 2:** Microstrain, residual compressive stress, coating thickness, interplanar spacing, and texture coefficient determined by XRD in an electrochemically deposited coating

Sample name	Crystallite size (nm)	Micro-strain *ε* (µε)	Calculated stress (MPa)	Coating thickness (µm)	Interplanar spacing (*d*) (nm)	Texture coefficient
						*I*_100_ *I*_111_ *I*_200_
Pristine Zn	26 ± 1	2.82 × 10^−6^	−0.21	85.4	1.707	7.21 37.11 43.64
Zn–TiO_2_	21 ± 3	2.56 × 10^−6^	−0.22	72.6	1.867	14.02 26.61 34.51
Zn–GrO	19 ± 5	1.76 × 10^−6^	−0.20	68.3	1.807	7.25 43.53 50.48
Zn–TiO_2_–GrO	18 ± 2	2.03 × 10^−6^	−0.25	64.0	1.513	10.48 19.11 43.42

The Zn–GrO and Zn–TiO_2_–GrO nanocomposite coatings show well-defined, flower-like surface features as illustrated in Fig. 1c and d. These morphologies develop as a result of favourable nucleation followed by regulated growth during the coating formation, and also due to their high electrical conductivity. The Backscattered electron micrographs (Fig. 1b and d) outline the uniform distribution of TiO_2_ particulates in the Zn matrix (Fig. 1b), in the interconnected GrO network (Fig. 1c) and hybrid graphene flakes with TiO_2_ embedded in the Zn matrix (Fig. 1d). Furthermore, the elemental composition of the coatings was confirmed by energy-dispersive X-ray spectroscopy (EDS) analysis, revealing Zn as the dominant constituent along with varying concentrations of Ti, O, and C corresponding to the incorporation of TiO_2_ and graphene oxide. The relatively high oxygen content detected by EDS, particularly in the pristine Zn coating, suggests the presence of oxygen-containing surface species in addition to metallic zinc. However, EDS is a surface-sensitive technique and cannot distinguish between metallic Zn and chemically bonded oxygen-containing phases. The detected oxygen may originate from a thin native ZnO passive layer formed upon exposure to air, adsorbed moisture, and oxygen-containing functional groups associated with graphene oxide. Although these oxygen-containing species contribute to the measured oxygen content, the XRD patterns remain predominantly characterised by metallic Zn reflections, indicating that the coating matrix is still largely Zn-rich. Therefore, the oxygen-rich species are considered to be confined mainly to the near-surface region rather than representing the dominant bulk phase of the coating.

The presence of sulfur in the EDS spectra is attributed to residual sulfate-containing species originating from the ZnSO_4_-based electrolyte used during electrodeposition. Although sulfur was detected in the coatings, no characteristic sulfate-related diffraction peaks were observed in the XRD patterns, indicating that sulfate-containing species do not constitute a distinct crystalline phase within the coating matrix and are primarily confined to the coating surface. Consequently, their contribution to bulk mechanical properties such as hardness and elastic modulus is expected to be negligible. Nevertheless, trace sulfate species may influence surface energy and wettability through polar interactions with water molecules at the coating surface. Similarly, their low concentration and surface-localised nature suggest only a marginal contribution to interfacial passivation and corrosion behaviour when compared with the dominant effects arising from coating microstructure, compactness, and the incorporation of TiO_2_ and graphene oxide reinforcements. Therefore, the overall structural, wettability, mechanical, and corrosion-related properties of the coatings are predominantly governed by the Zn matrix and the incorporated reinforcement phases rather than sulfate-derived surface residues.

The detection of carbon in the Zn–GrO and Zn–TiO_2_–GrO coatings confirms the successful incorporation of graphene oxide into the Zn matrix. The lower carbon content observed in the Zn–TiO_2_–GrO coating compared with the Zn–GrO coating may be attributed to the simultaneous incorporation of TiO_2_ particles and the heterogeneous distribution of reinforcement phases within the metallic matrix. Furthermore, EDS is a semi-quantitative technique and exhibits lower sensitivity towards light elements such as carbon than towards heavier metallic elements. Therefore, the measured carbon concentration should be considered as evidence of graphene oxide incorporation rather than an absolute representation of its content within the coating.^18^

**Fig. 2:**
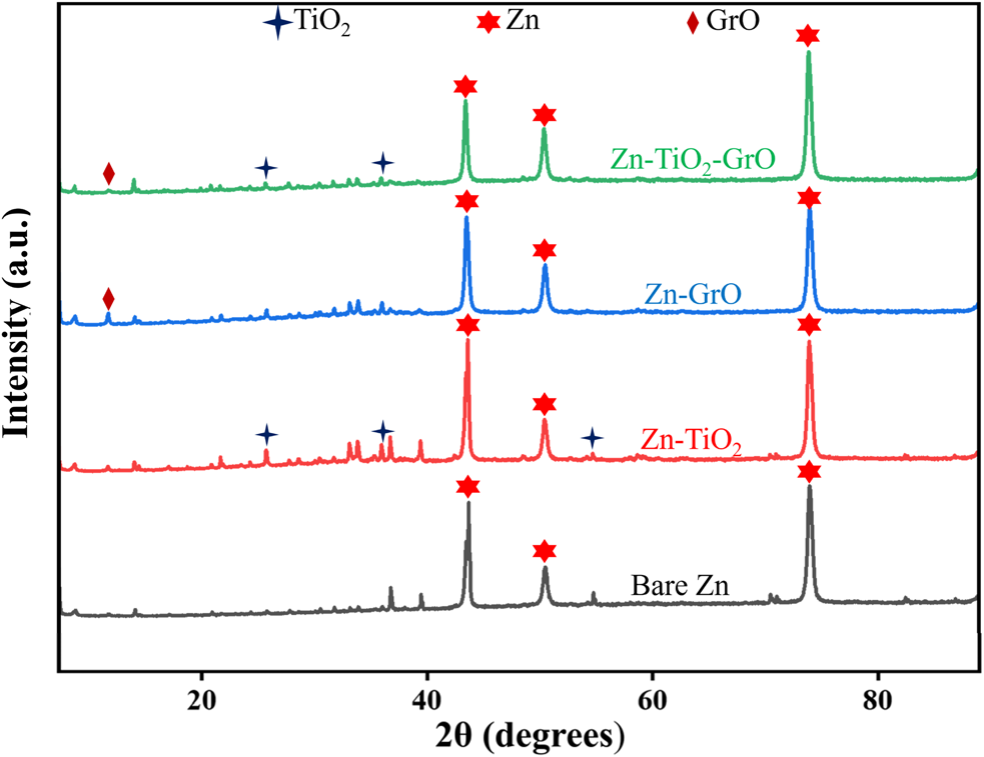
XRD pattern of Zn and Zn-composite coatings.

The X-ray diffraction technique was also performed to examine the zinc (Zn)-based composite coatings illustrated in Fig. 2. The diffraction spectra confirmed the successful electrodeposition of Zn coating at (101), (102), and (110) planes of hexagonal Zn, observed at 2*θ* angles of approximately 43.5°, 54.3°, and 70.7°, respectively.^19^ In the Zn–GrO composite, additional reflections attributable to GrO are discernible, notably at 10.34° corresponding to the (001) plane, consistent with the characteristic interlayer spacing of GrO.^20,21^ Furthermore, additional diffraction peaks associated with TiO_2_ were observed in the Zn–TiO_2_ and Zn–TiO_2_–GrO coatings, indicating the successful incorporation of TiO_2_ particles at 36.45° (002) and 27.93°, confirming the multiphase composition of the composite layers.^19^ The 27.93° peak likely arises from TiO_2_ anatase (110) phase.^22^

Crystallite size was calculated by using the Scherrer eqn (1), showing, Zn–TiO_2_–GrO coating exhibited the smallest average crystallite size of (18 ± 2 nm). This refinement is primarily attributed to the homogeneous dispersion and effective grain refinement due to the combined incorporation of TiO_2_ and GrO. In contrast, the Zn–GrO composite showed slightly larger crystallites (19 ± 5 nm), likely due to less effective grain growth suppression owing to the absence of TiO_2_'s grain refining influence. Significantly larger crystallite sizes were noted for the Zn–TiO_2_ (21 ± 3 nm) and pristine Zn (26 ± 1 nm) coatings, which can be ascribed to enhanced grain coarsening driven by the absence or reduced nanoparticle dispersion and higher tendency for agglomeration of Zn and TiO_2_ particles (Table 2). This agglomeration reduces grain boundary area and promotes crystal growth, resulting in coarser grains within the composite matrix. The texture coefficient (*I*_tc_) calculated from the eqn (2) for all the coatings with respect to the planes (111), (200), and (220) is summarised in Table 2.2
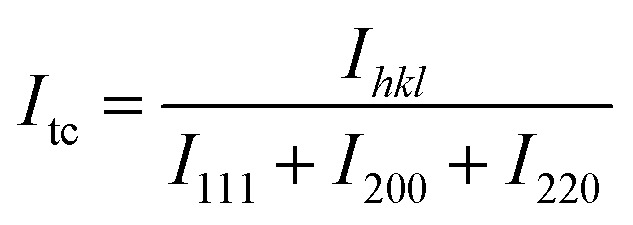


The lattice microstrain produced in the coated systems was measured, and the size and strain-induced widening of the XRD peaks were calculated using the Williamson–Hall (W–H) eqn (2).3
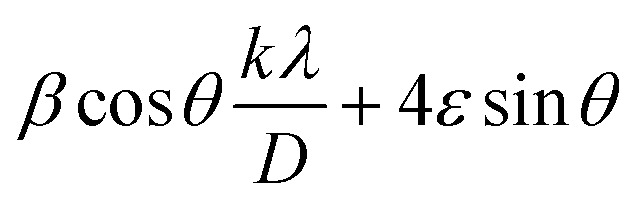
where *β* is the Full Width at Half Maximum (FWHM) in radians, *θ* is the Bragg diffraction angle, *K* is the shape factor (0.9), *λ* is the X-ray wavelength (0.154 nm for Cu Kα radiation), *D* is the crystallite size, and *ε* is the micro-strain.

**Fig. 3:**
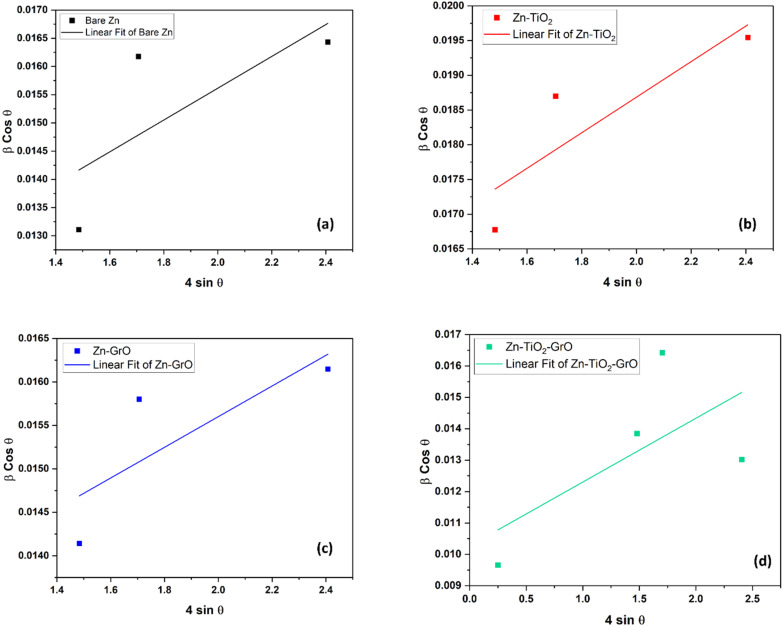
Williamson–Hall plots of (a) bare Zn, (b) Zn–TiO_2_, (c) Zn–GrO, and (d) Zn–TiO_2_–GrO coatings.

A plot of *β* cos *θ versus* 4 sin *θ* in Fig. 3 yields a straight line in which the intercept provides the crystallite size contribution (*kλ*/*D*), while the slope gives 4*ε*. The microstrain was therefore obtained as4
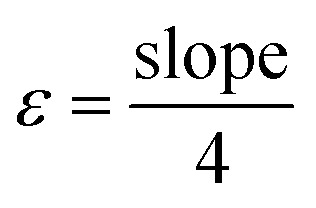


The extracted *ε* values for all coatings fall in the range of 1.7–2.8 µε (Table 2), indicating only very small lattice distortions. To convert these microstrain values into residual stress, *ε* was first transformed into unitless strain (*ε* × 10^−6^) and then substituted into the X-ray elastic constant relation for a biaxial stress state:5
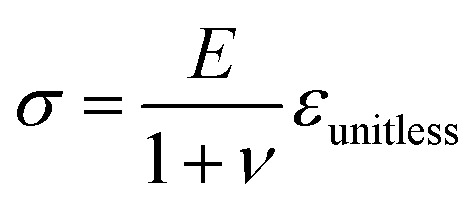
where *E* and *ν* are the elastic modulus and Poisson's ratio of the coatings, respectively. Based on this formulation, the calculated residual stresses were found to be −0.21 MPa (pristine Zn), −0.22 MPa (Zn–TiO_2_), −0.20 MPa (Zn–GrO), and −0.25 MPa (Zn–TiO_2_–GrO), confirming that all coatings exhibit very small compressive stresses as depicted in Table 2. Because it prevents crack formation and improves mechanical adherence to the substrate, such compressive residual stress is advantageous for coating integrity.^23^ Additionally, the tiny amount of tension indicates that there is no substantial mechanical deformation or thermal mismatch introduced during the fabrication process.

Since the W–H microstrain values lie only in the microstrain (10^−6^) range, the resulting stresses are expected to be in the sub-MPa level, rather than the hundreds of MPa typically associated with plastically deformed or thermally shocked coatings. The slight compressive character suggests a slight lattice contraction, which may be caused by the addition of smaller ionic species, interfacial constraint by the substrate, or by the effect of nanocrystalline packing. Significantly, these small compressive stresses are desirable since they inhibit crack formation and increase the coating bonding, but also imply that there was no harmful thermal or mechanical incompatibility during processing. Therefore, the W–H analysis is able to not only measure the microstrain but also ensure the structural stability of the Zn-based composite coatings. The apparent presence of TiO_2_-related peaks in Zn–GrO and GrO-related features in Zn–TiO_2_ coatings can be attributed to peak overlap, low-intensity signals due to the low reinforcement fraction, and peak broadening associated with nanoscale crystallinity. Such effects may lead to minor spectral contributions that appear across different composite systems, even in the absence of direct phase incorporation.

Compressive stress is present in the coatings, showing the negative residual stress values generated from the W–H-derived microstrain (Fig. 3). After the addition of TiO_2_ and graphene oxide, the diffraction peaks of Zn showed a slight shift to larger 2*θ* angles, which means that the lattice parameter of the material was slightly contracted, and low compressive residual stress was produced (−0.20 to −0.25 MPa). These compressive stresses are usually useful for the integrity of the coating since they can suppress crack formation and enhance the stability of the coating. The relatively low stresses obtained indicate that there was minimal distortion of the Zn matrix introduced when the nanoparticles were incorporated. The Zn–TiO_2_–GrO coating shows slightly higher compressive stress, which could be explained by the synergistic effect of TiO_2_ nanoparticles and graphene oxide on the packing density of the lattice and the interactions between the interfaces in the coating.^24^

**Fig. 4:**
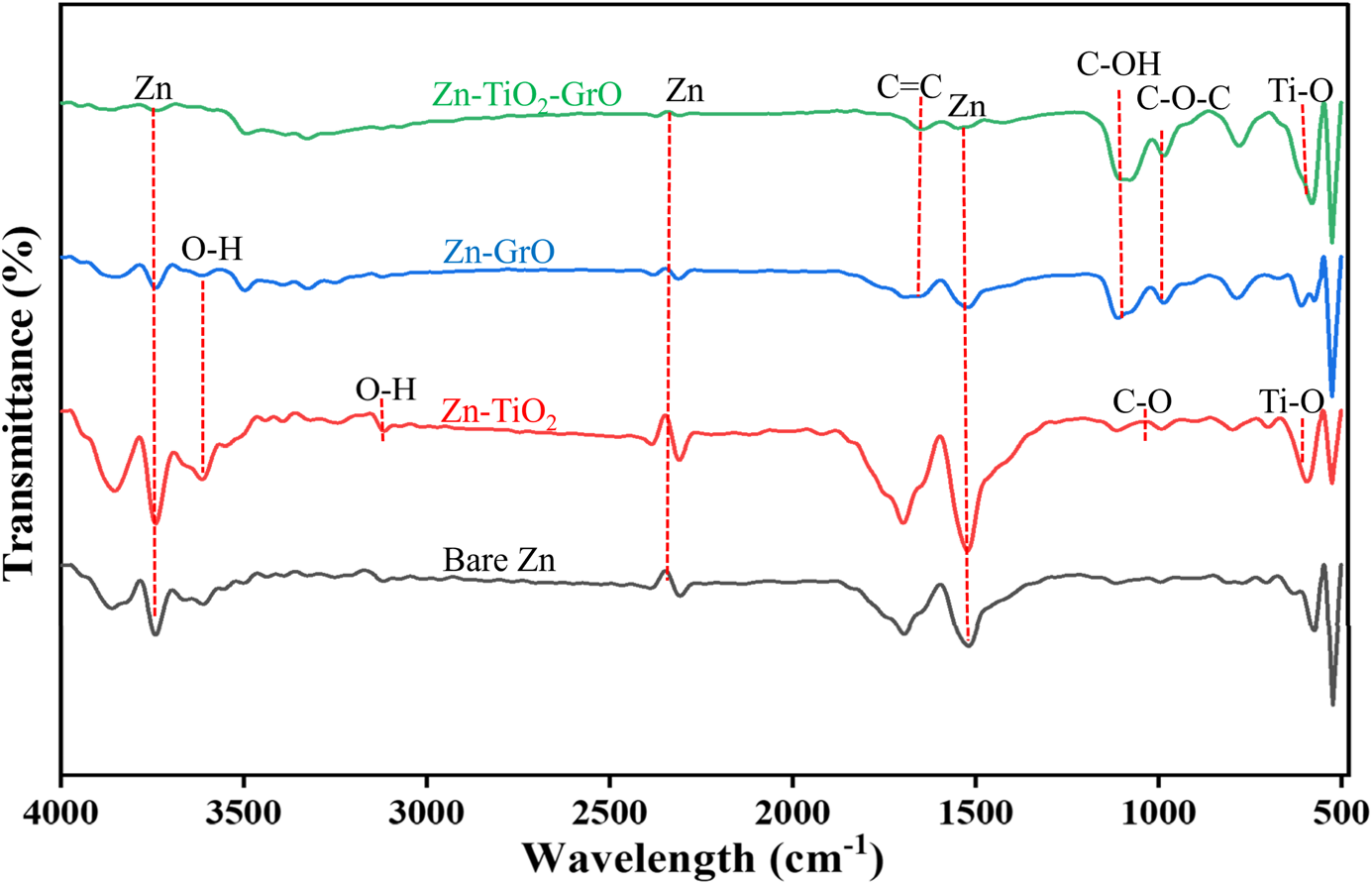
FTIR spectra of pristine Zn and Zn composite coatings.

FTIR spectra show the asymmetric stretching vibrations of hydroxyl (C–OH) at 1400 cm^−1^.^25^ The band observed near 1550 cm^−1^ is attributed to C=C stretching vibrations associated with GrO. The broad absorption band in the range of 3200–3700 cm^−1^ corresponds to –OH stretching vibrations from hydroxyl groups and adsorbed moisture. Zn–O lattice vibrations are typically observed in the lower wavenumber region, consistent with the reported literature.^26^ Other absorption bands that emerged at 1550 cm^−1^, 2375 cm^−1^, and 3700 cm^−1^ were typical of ZnO-related stretching modes and hydroxyl (–OH) groups.^27^ The broad band centred near 3375 cm^−1^ corresponds to –OH stretching vibrations arising from surface-adsorbed water molecules or hydroxyl groups present on the Zn–TiO_2_ surface. A distinct band at 1606 cm^−1^ was assigned to C=C stretching vibrations, which are primarily associated with the sp^2^-hybridized carbon framework of graphene oxide or residual carbonaceous species within the composite matrix.^28^ Bands were observed at 1091 cm^−1^ and 1150 cm^−1^, which were attributed to C–O and C–OH vibrations, respectively. Additionally, the absorption band around 1050 cm^−1^ was attributed to the C–O–C stretching mode of epoxy groups. The TiO_2_ lattice vibrations typical of TiO_2_ were strong at frequencies around 600 cm^−1^, which is indicative of the metal–oxygen bonding within the TiO_2_ crystal structure (Fig. 4).

### 3.2 Hydrophilicity and surface roughness of Zn composite coatings

**Fig. 5:**
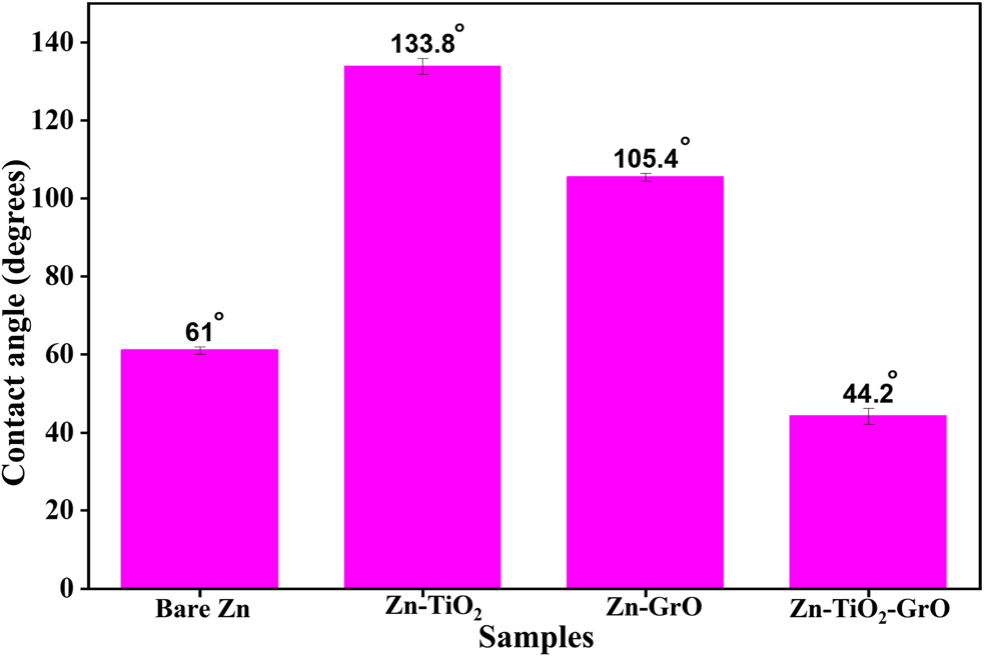
Measurement of the static contact angle of pristine Zn and Zn composite coating.

Out of the four Zn-based electrodeposited coatings, the first three samples exhibit poor wettability performance as shown in Fig. 5 because of the mismatched surface roughness-contact angle (OCA) relationships that undermine corrosion protection and functional versatility. In contrast, the pristine Zn coating shows excellent performance with slight topographic-wettability synergy. The Zn–TiO_2_ coating exhibited a relatively high contact angle (133.8 ± 1.2°) with a surface roughness of 0.484 ± 0.01 µm, indicating enhanced hydrophobic behaviour compared with the pristine Zn coating. The pristine Zn coating (*R*_a_ = 0.882 ± 0.03 µm, *θ* = 61 ± 0.3°) has a very rough and hierarchically structured surface and moderate hydrophilicity, summarised in the Wenzel wetting regime (Table 3). In this regime, when the surface roughness is higher (*r* > 1.5), the liquid enters the surface asperities and becomes trapped in localised reservoirs within the electrolyte. These entrapped electrolytes can cause galvanic corrosion to occur rather than to prevent it or to provide uniform sacrificial protection.

**Table 3:** The data of hardness, surface roughness and contact angle of Zn-based coatings. Values are the mean ± SD

Sample name	Rockwell hardness (HRB)	Vickers hardness (HV)	Surface roughness (µm)	Contact angle (degrees)
Pristine Zn	41 ± 3.2	17 ± 1	0.882 ± 0.03	61 ± 0.3
Zn–TiO_2_	64 ± 2.3	19 ± 2	0.484 ± 0.01	133.8 ± 1.2
Zn–GrO	71 ± 1.2	24 ± 1	0.544 ± 0.04	105.4 ± 2.3
Zn–TiO_2_–GrO	75 ± 3.4	29 ± 2	0.157 ± 0.06	44.2 ± 0.2

The trapped air layer is therefore vulnerable to mechanical shear or vapour condensation, which decreases long-term electrolyte exclusion, important for marine exposure conditions.^29^ Likewise, the Zn–GrO coating (*R*_a_ = 0.544 ± 0.04 µm, *θ* = 105.4 ± 2.3°) has an intermediate hydrophobic property, which is not ideal, since the surface roughness is not sufficient to enhance the intrinsic non-wettability. This restricts its use for long-term corrosion protection and adhesion-based use, like automotive primer layers.^30^ The Zn–TiO_2_–GrO hybrid coating, however, has a significantly smoother surface (*R*_a_ = 0.157 ± 0.06 µm, *θ* = 44.2 ± 0.2°) and exhibits much better performance. The reduction in roughness results in more uniform spreading of the liquid (*θ* < 50°), suggesting that the liquid behaviour is closer to the Young regime (*r* ≈ 1.05). The low level of surface irregularities allows the formation of a continuous and homogeneous liquid film, especially beneficial for the strong and durable adhesion of the coating to the surface in the automotive and architectural fields.

**Fig. 6:**
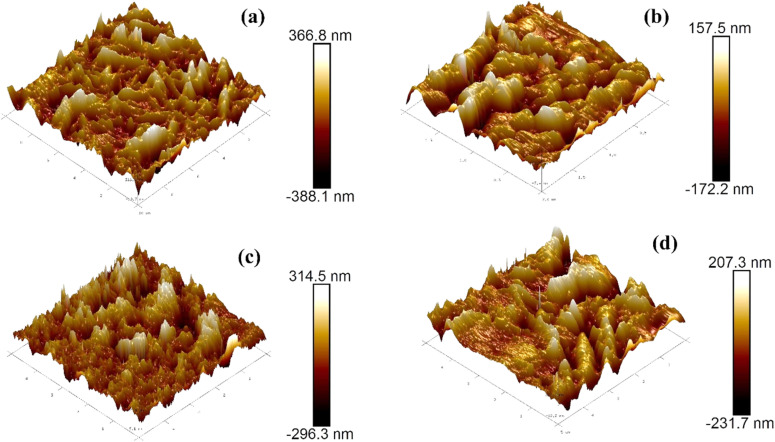
AFM 3D images of Zn composite coatings are presented in (a) Pristine Zn, (b) Zn–TiO_2_, (c) Zn–GrO, and (d) Zn–TiO_2_–GrO, respectively.

The nanoscale differences among the coatings are also revealed by atomic force microscopy (AFM), which also shows the positive effect of hybrid reinforcement. The surface of the pristine Zn coating is very rough, with irregular platelet-like crystallites, as evidenced by high *R*_a_, *R*_q_, and *R*_max_ values of 72.6 nm, 97.1 nm, and 868 nm, respectively. The above features are characteristics of uncontrolled Zn grain growth during electrodeposition and can affect coating integrity. The incorporation of TiO_2_ nanoparticles greatly improves the surface morphology, resulting in a more compact and homogeneous structure and lower surface roughness (*R*_a_ = 38.9 nm). The Zn–GrO coating has moderate roughness and surface heterogeneity (*R*_a_ = 59.0 nm, *R*_q_ = 78.7 nm, *R*_max_ = 668 nm), because of partial agglomeration of GrO sheets and non-uniform grain development. The hybrid coating of Zn–TiO_2_–GrO, however, has an optimised and well-balanced surface morphology with relatively low values of roughness (*R*_a_ ≈ 42 nm, *R*_q_ ≈ 56 nm, *R*_max_ ≈ 518–534 nm) and a hierarchical nanostructure. The surface is slightly negatively skewed (−0.286) and has a high kurtosis (∼4.25), suggesting a surface with well-distributed valleys and controlled topographical features, as shown in Fig. 6. The synergistic interaction between TiO_2_ nanoparticles and graphene oxide leads to this optimised morphology, which provides better dispersion of particles, reduces agglomeration and increases the connectivity of the interfaces. As a result, the coating of Zn–TiO_2_–GrO has excellent surface properties, which are especially suitable for applications where controlled wettability, better adhesion, larger active surface area and better functional properties are required. This topographic smoothness facilitates rapid electrochemical activation by ensuring uniform electrolyte access across the coating surface, optimising sacrificial anode efficiency in chloride-rich environments through consistent Zn dissolution kinetics without roughness-trapped dead zones that impede galvanic protection. Furthermore, the hydrophilic character promotes sheeting self-cleaning *via* thin water film flow that efficiently sweeps particulates, salts, and atmospheric contaminants, preventing pitting initiation delivering balanced topography-wettability synergy unmatched by rougher composites, as the synergistic TiO_2_–GrO heteroaggregation compacts lamellar microstructures, exposing abundant –OH groups from anatase facets and GrO edges that overwhelm intrinsic Zn polarity to enforce complete wetting despite nanoparticle presence, corroborated by systematic OCA-roughness correlations in Zn composite electrodeposits reported Tang *et al.*^31^

### 3.3 Mechanical hardness and theoretical modelling

#### 3.3.1 Experimental hardness evaluation

The Rockwell and Vickers hardness tests were used to assess the mechanical properties of the coatings. Table 3 shows that the coating with the highest hardness values was Zn–TiO_2_–GrO (75 HRB and 29 HV), while the pristine Zn coating showed the lowest values (41 HRB and 17 HV). The incorporation of TiO_2_ and graphene oxide significantly improved the hardness of the Zn matrix, with Zn–TiO_2_ (64 HRB, 19 HV) and Zn–GrO (71 HRB, 24 HV) coatings showing intermediate performance.

The synergistic reinforcement effect of TiO_2_ nanoparticles and graphene oxide sheets might be responsible for the better hardness of the Zn–TiO_2_–GrO coating. These reinforcements are uniformly distributed throughout the volume, which helps to refine the grain, inhibits dislocation movement and enhances stress transfer through the coating matrix, as verified by Williamson–Hall analysis. As a result, the hybrid coating is more resistant to localised plastic deformation and has a better load-bearing capacity. The results of the present study show that the composition of the Zn–TiO_2_–GrO coating offers the highest strength. Moreover, the Vickers hardness value (29 HV) is higher than the values of several composite coatings based on Zn, which are reported in the literature, thus demonstrating the positive impact of the hybrid reinforcement strategy. For example, Tafreshi *et al.*^32^ synthesised Zn–Ni alloy coatings *via* electrodeposition with systematically varied nickel content within the zinc matrix, exhibiting microhardness values in the range of 48 HRB. The high Rockwell hardness of Zn–TiO_2_–GrO coating, though not easily comparable against other hardness scales, suggests a mechanical performance comparable to and possibly higher than several reported Zn-based alloy and composite coatings.

#### 3.3.2 Theoretical evaluation of hardness based on micromechanical models

The theoretical hardness was determined with the help of the elastic-mechanical correlations derived based on the measured Young's modulus and Poisson ratio to better understand the mechanical response of the Zn-based composite coatings. Theoretical hardness is applicable in the study of the intrinsic load-bearing capacity and strengthening mechanisms of composite coating, especially when experimental nanoindentation is affected by surface roughness, porosity, and substrate effects. Thus, predictive models founded on elastic constants were used to get a good approximation of intrinsic hardness. In order to have a complete assessment, three theoretical models-Tabor, Pugh, and Chen-Niu were used. These models relate indentation hardness to key elastic parameters such as Young's modulus or Elastic modulus (*E*), shear modulus (*G*), and bulk modulus (*B*). The combined use of these models provides upper, lower, and refined estimates, yielding a more accurate prediction of the mechanical behaviour of the coatings.

##### 3.3.2.1 Tabor relation (elastic modulus-based hardness)

Tabor theorised that hardness (*H*) is approximately proportional to the Young's modulus of the material. This will provide a first-order upper bound on the theoretical hardness, particularly for metallic and metal-oxide coatings. The Tabor relation is not a complex one and is simply common in the analysis of hardness, and is the most common reference of comparison. Based on the classical relation by Tabor, the ratio of the hardness (*H*) to the yield strength (*Y*) is about (*H*/*Y* ≈ 3). Because with thin coatings, it is difficult to directly measure *Y*, an elastic-modulus-based approximation (*H* ≈ 0.07*E*) is often employed in coating literature as an expedient simplification of the Tabor model.

6 *H* ≈ 0.07*E*

where: *H* = theoretical hardness (GPa).

##### 3.3.2.2 Hardness from shear modulus (*G*)-Pugh relations

Pugh demonstrated that the ratio of the shear modulus (*G*) to the bulk modulus (*B*) governs ductile–brittle behaviour, and *G* itself directly correlates with hardness. The shear modulus is computed from the measured elastic modulus and Poisson's ratio (*v*): 7
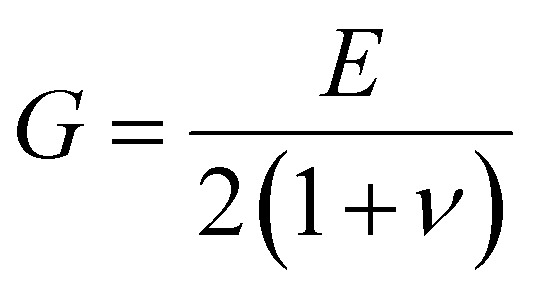


The corresponding theoretical Vickers hardness is estimated using the widely applied shear-modulus-based relation:

8 *H*_v_ = 0.151*G*

##### 3.3.2.3 Hardness from bulk & shear modulus (Chen model)

To obtain a more refined and physically representative hardness estimate, the Chen-Niu empirical model was applied. This model incorporates both bulk modulus (*B*) and shear modulus (*G*), capturing the combined resistance to uniform and shear deformation:

9 *H*_v_ = 2(*k*^2^*G*)^0.585^ − 3

where,10
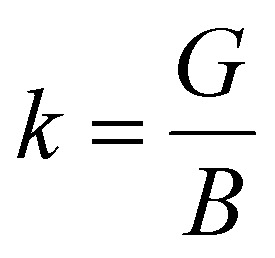
and the bulk modulus is calculated from elastic constants:11
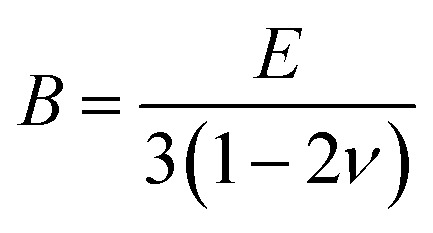


The Chen model is widely recognised for accurately describing the hardness of metal-ceramic and oxide-reinforced nanocomposites.

##### 3.3.2.4 Comparison of theoretical hardness models

**Fig. 7:**
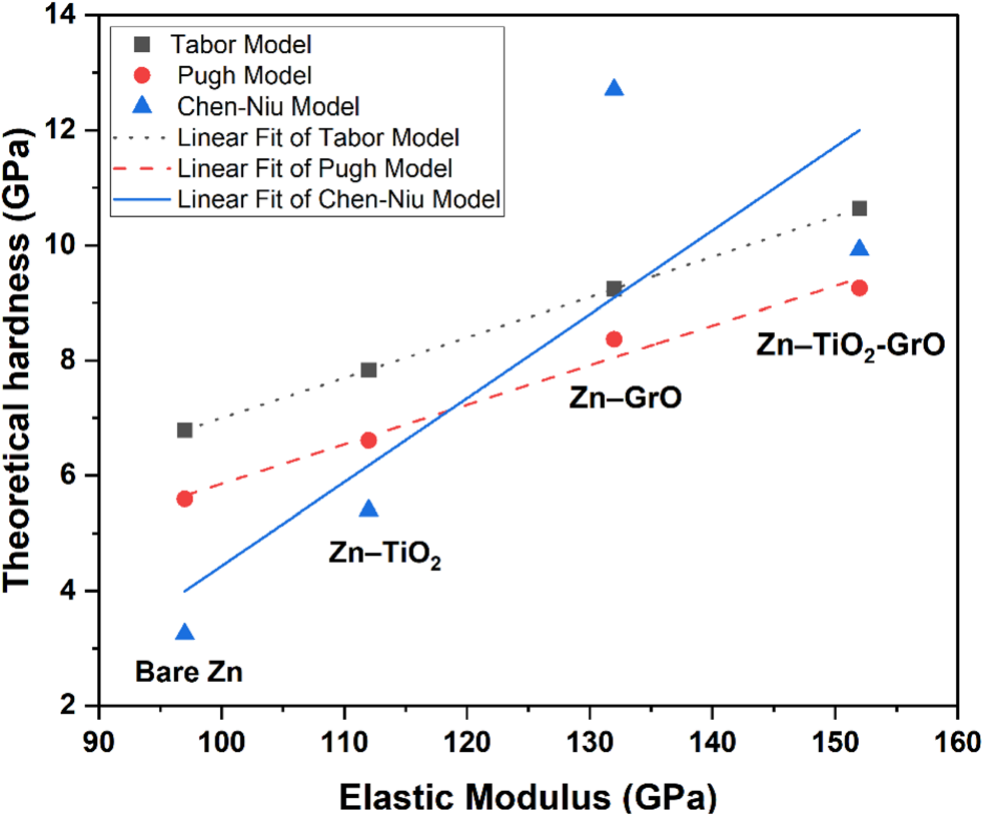
Correlation between elastic modulus and theoretical hardness predicted using three different models Tabor, Pugh, and Chen-Niu for Pristine Zn, Zn–TiO_2_, Zn–GrO, and Zn–TiO_2_–GrO coatings.

Through all these strategies, the theoretical hardness of the composite coatings is increasing with the incorporation of TiO_2_ and graphene oxide (GrO). According to Tabor and Pugh's estimations, there is a progressive increase in stiffening of pristine Zn (6.79 and 5.59 GPa, respectively) to that of the Zn–TiO_2_–GrO hybrid (10.64 and 9.25 GPa, respectively), of elastic and shear rigidity with reinforcement. The Chen model yields several different magnitudes, with the most significant differences in Zn–GrO (12.7 GPa) due to the ratio of shear to bulk moduli (*k* = *G*/*B*) being a key factor. High G/B ratios enhance the Chen prediction of hardness, as shown in Table 4. These differences between models are normal and informative: Tabor presents a simple modulus-scaled baseline, Pugh emphasises shear-limited plasticity, and Chen presents an optimistic prediction which is generally more precise in mixed metal-ceramic systems, as shown in Fig. 7.

**Table 4:** Theoretical hardness estimates for Zn-based coatings calculated using measured elastic constants

Sample name	*E* (GPa)	*ν*	*G* (GPa)	*B* (GPa)	*H*_Tabor_ (GPa)	*H*_Pugh_ (GPa)	*H*_Chen_ (GPa)
Pristine Zn	97	0.31	37.0229	85.0877	6.79	5.59	3.25
Zn–TiO_2_	112	0.28	43.7500	84.8485	7.84	6.61	5.40
Zn–GrO	132	0.19	55.4622	70.9677	9.24	8.37	12.70
Zn–TiO_2_–GrO	152	0.24	61.2903	97.4359	10.64	9.25	9.92

Practical implications are twofold. To begin with, it is universally observed in all models that the theoretical hardness is always increased when TiO_2_ and GrO are added, which supports the experimental prediction that the hardness would increase because of rigid particle/platelet reinforcement and hindering the movement of dislocations at matrix/filler interfaces. Second, discrepancies among model predictions highlight the importance of microstructural effects such as interface quality, porosity, anisotropy, and load-transfer efficiency that are not captured by simple homogenization approaches. Hence, the theoretical values should be treated as predictive bounds and compared with elastic modulus values estimated using the Chen model and theoretical homogenization approaches.

#### 3.3.3 Rule-of-mixtures (ROM) analysis of elastic modulus

The Rule of Mixtures (ROM) was utilised to theoretically introduce the effective elastic characteristics of Zn-based composite coatings by considering the contributions of TiO_2_ and GrO reinforcements into account. ROM is a first-order method to estimate the composite stiffness, which assumes that the overall property is controlled by the volume-weighted behaviour of each individual constituent phase. Whereas ROM does not consider microstructural mechanisms, like agglomeration of particles, interfacial bonding, or distribution of defects, it is a popular predictive model of metallic and ceramic-reinforced nanocomposites. Both the Voigt (iso-strain) and Reuss (iso-stress) models were used in obtaining a comprehensive upper and lower theoretical bound. The Voigt model assumes that strain behaviour in all phases is the same and is the maximum likely modulus of well-bonded and uniformly dispersed reinforcements. By comparison, the Reuss model, where stress is assumed to be equal in all phases, is the model with the least expected modulus, particularly in cases where the load transfer between reinforcements and the matrix is weak, or agglomeration takes place. The average of the two bounds is termed the Hill average, which is taken as the most realistic estimate of heterogeneous electrodeposited nanocomposite coatings.

##### 3.3.3.1 Voigt model (upper bound, iso-strain condition)

The Voigt model is based on the assumption that the loading process exerts identical strain on the matrix and the reinforcement phases. This is the upper bound of the composite property and is normally applicable where the reinforcement is stiff, dispersed well and firmly stuck on the matrix.

12 *E*_V_ = *V*_m_*E*_m_ + Σ*V*_r_*E*_r_

where *E*_V_ = Voigt modulus, *V*_m_, *V*_r_ = volume fractions of matrix and reinforcements, *E*_m_, *E*_r_= Modulus of matrix and reinforcements.

This model reflects the perfect examples of strong interfacial adhesion and total transfer of loads, which is anticipated in cases when rigid nanoparticles are evenly distributed.

##### 3.3.3.2 Reuss model (lower bound, iso-stress condition)

The Reuss model assumes that the phase stress is the same. This gives the lower bound and is when the reinforcement allocation is not ideal or when the load transfer efficiency is extremely low.13
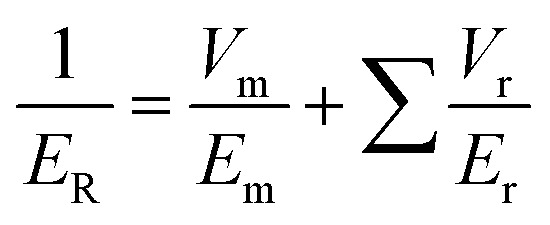
where *E*_R_ = Reuss modulus.

This lower limit is useful for understanding the minimum expected property in coatings with imperfect bonding or agglomeration.

##### 3.3.3.3 Hill average (realistic estimate)

Since both Voigt and Reuss are inaccurate models of real composite behaviour, we frequently use the Hill average, the arithmetic average of the two, as the most physically realistic theoretical model:14
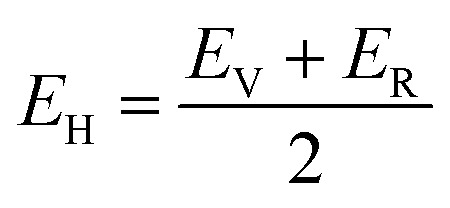
where: *E*_H_ = Hill modulus.

This hybrid prediction is commonly used in high-impact materials science publications to present a balanced estimate of composite stiffness.

##### 3.3.3.4 ROM predictions and interpretation

**Fig. 8:**
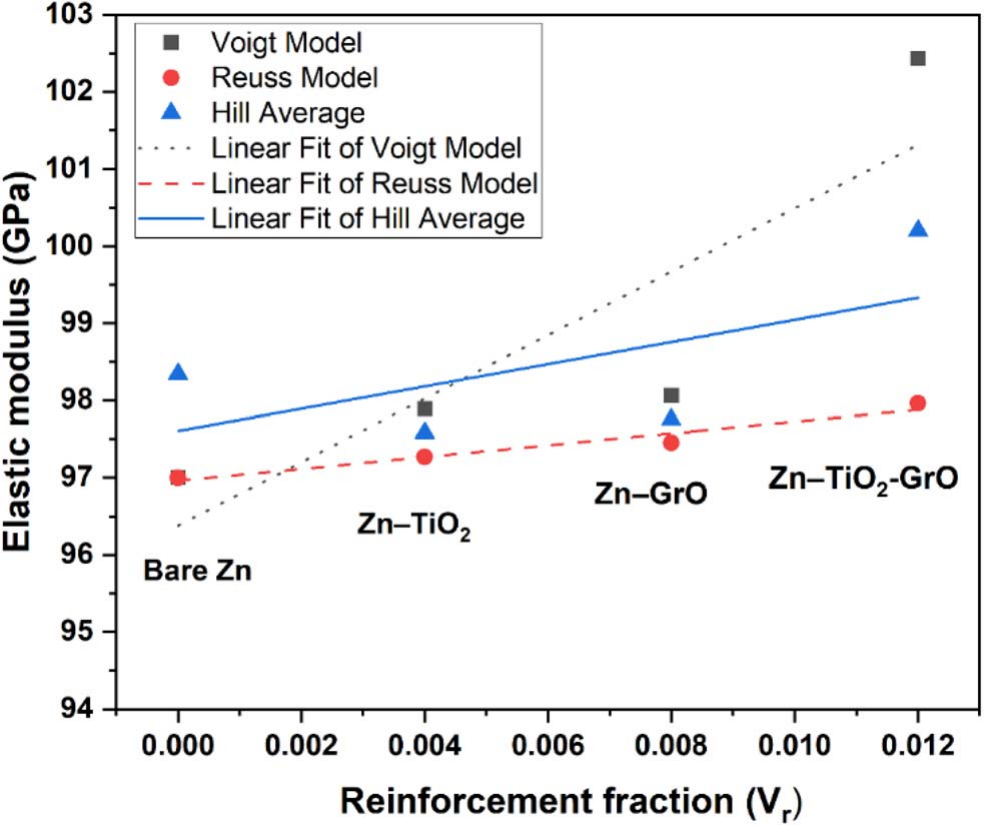
ROM prediction of elastic modulus as a function of reinforcement volume fraction (*V*_r_) for Zn, Zn–TiO_2_, Zn–GrO and Zn–TiO_2_–GrO coatings.

According to the ROM analysis, the elastic moduli of all fractions of reinforcements are predicted using the Voigt, Reuss, and Hill models and are near the intrinsic modulus of Zn since the reinforcement content is very low (=1.2 vol%). The average values of the hills are obtained from Zn–TiO_2_ (97.757 GPa), Zn–GrO (97.58 GPa), and Zn–TiO_2_–GrO (100.20 GPa), indicating only a marginal theoretical increase in stiffness relative to pristine Zn (97 GPa) (Table 5). This proves that in this case of low reinforcement fractions, the strength of the composite is dominated by the Zn matrix, and the linear ROM assumptions mean little enhancement. Nonetheless, when comparing them with experimentally determined values of the elastic modulus (far larger than those predicted by simple linear mixing in the real data), a clear indication is given that simple linear mixing is not entirely capable of explaining the mechanical behaviour of such coatings. The fact that it does not follow the ROM indicates that there are other strengthening mechanisms, which include refined microstructure, good Zn-reinforcement interfacial bonding, transfer of loads, better packing density, and even the formation of the graphene-oxide network, all of which increase the rigidity beyond the ROM predictions. Thus, the experimentally measured values are the contribution of microstructural effects and reinforcement synergy that is difficult to explain by simple idealised mixing models, whereas the ROM results are a theoretical lower-bound estimate of the same. Fig. 8 demonstrates the deviation of elastic modulus as a function of reinforcement volume fraction (*V*_r_) of the Zn-based composite coatings, as per the Voigt, Reuss, and Hill models. The Voigt model (upper bound) provides the maximum modulus predicted by its assumption of iso-strain conditions, and the Reuss model (lower bound) provides the lowest theoretical modulus predicted by an iso-stress assumption. The Hill average, or the arithmetic mean of the Voigt and Reuss bounds, falls in between the two and gives the most realistic approximation to the case of randomly dispersed reinforcements. All three theoretical predictions depict a slight positive trend with the increase in *V*_r_ between 0 to 0.012, which is in line with the low reinforcement fractions. The data points of the plotted graphs of each sample (Pristine Zn, Zn–TiO_2_, Zn–GrO, and Zn–TiO_2_–GrO) indicate that the theoretical values of Voigt, Reuss, and Hill are concentrated around the values of 97–100 GPa. This verifies that at small *V*_r_, the Zn matrix is the most important in determining the total stiffness, and the ROM only predicts an incremental change in the elastic modulus. Linear fittings incorporated in each model also indicate a small yet positive slope of the modulus with the increase in *V*_r_.

**Table 5:** Theoretical elastic modulus bounds for Zn and Zn-based composite coatings using Voigt (iso-strain upper), Reuss (iso-stress lower), and Hill average models

Sample name	Matrix modulus (*E*_m_) (GPa)	Reinforcement modulus (*E*_r_) (GPa)	Reinforcement fraction (*V*_r_)	Matrix volume (*V*_m_)	Voigt upper bound (*E*_V_)	Reuss lower bound (*E*_R_)	Hill average (*E*_H_)	*E*_exp_ (GPa)	Reinforcement factor (*R*_f_)
Pristine Zn	97	—	0.000	1.000	97	97	98.340	97.0	—
Zn–TiO_2_	97	230	0.008	0.992	98.064	97.451	97.757	112.0	23.7
Zn–GrO	97	320	0.004	0.996	97.892	97.271	97.581	132.0	55.9
Zn–TiO_2_–GrO	97	550	0.012	0.998	102.43	97.96	100.20	152.0	12.1

##### 3.3.3.5 ROM-based reinforcement factor

To further quantify the deviation of experimental elastic modulus from theoretical ROM bounds, a reinforcement factor (*R*_f_) was calculated using the normalized difference between the experimental modulus and the Reuss lower bound:15
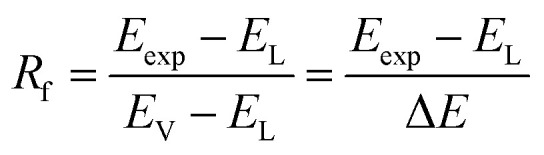
where *E*_exp_ = experimentally measured elastic modulus, *E*_V_ = Voigt (upper bound), *E*_L_= Reuss (lower bound), Δ*E* = *E*_V_ − *E*_L_.

Since *R*_f_ is sensitive to the width of the ROM interval, particularly at low reinforcement fractions the calculated values are interpreted qualitatively to assess the extent of reinforcement effectiveness.

*R*_f_ = 0 when the experimental modulus coincides with the Reuss bound and 1 when it coincides with the Voigt bound; values >1indicate an experimental modulus exceeding the Voigt prediction. Using the ROM bounds from Table 5, the computed reinforcement factors were 23.7 (Zn–TiO_2_), 55.9 (Zn–GrO), and 12.1 (Zn–TiO_2_–GrO) as shown in Table 5. For pristine Zn, *R*_f_ is not defined because *E*_V_ = *E*_L_. The higher *R*_f_ value observed for Zn–GrO suggests a stronger contribution of graphene oxide to the calculated reinforcement response. However, the calculated reinforcement factor indicates the contribution of the incorporated reinforcements to the mechanical response of the coatings. Owing to its sensitivity to input parameters and reinforcement volume fraction, *R*_f_ should be considered as a qualitative indicator rather than an absolute measure of reinforcement efficiency. Therefore, the overall performance of the coatings should be interpreted in conjunction with the experimental hardness, surface roughness, wettability, and microstructural results.

## 4. Conclusion

This study demonstrates that the co-electrodeposition of TiO_2_ nanoparticles and graphene oxide within a Zn matrix provides an effective strategy for engineering multifunctional Zn-based composite coatings. The composite coatings exhibited enhanced surface and mechanical properties. The hydrophilicity, surface roughness, hardness and elastic modulus were found to be improved for Zn–TiO_2_–GrO composite coating (*θ* = 44.2°, *R*_a_ = 0.157 µm, 29 HV, and 9.92 GPa, respectively) when compared to other fabricated coatings. These significantly amended properties were attributed to the synergistic effect and the homogeneous dispersion of TiO_2_ and GrO additives, which offered grain refinement and strong interfacial interactions. The experimental mechanical performance of the coatings was further compared with theoretical values using various micromechanical models, including the rule of mixtures (ROM), the combined Voigt–Reuss (V–R) and Hill model for upper and lower bounds. Overall, the Zn–TiO_2_–GrO emerges as a promising composite coating with potential applications in biomedical devices, photocatalysis, energy systems, and other advanced industrial applications.

## Conflicts of interest

The authors declare no conflict of interest.

## Data Availability

Data will be made available on genuine request.

## References

[cit1] Wang Z. L. (2008). Splendid One-Dimensional Nanostructures of Zinc Oxide: A New Nanomaterial Family for Nanotechnology. ACS Nano.

[cit2] Gulab H., Fatima N., Tariq U., Gohar O., Irshad M., Khan M. Z., Saleem M., Ghaffar A., Hussain M., Khaliq Jan A., Humayun M., Motola M., Hanif M. B. (2024). Advancements in zinc oxide nanomaterials: Synthesis, properties, and diverse applications. Nano-Struct. Nano-Objects.

[cit3] Rahman W., Garain S., Sultana A., Ranjan Middya T., Mandal D. (2018). Self-Powered Piezoelectric Nanogenerator Based on Wurtzite ZnO Nanoparticles for Energy Harvesting Application. Mater. Today: Proc..

[cit4] Awasthi S., Prior Palomero B., Srivastava A., Selvaraj S., Pandey S. K. (2024). Nanodiamond-structured zinc composite coatings with strong bonding and high load-bearing capacity. Nanoscale Adv..

[cit5] Antony Jose S., Lapierre Z., Williams T., Hope C., Jardin T., Rodriguez R., Menezes P. L. (2025). Wear- and Corrosion-Resistant Coatings for Extreme Environments: Advances, Challenges, and Future Perspectives. Coatings.

[cit6] Xiao N., Tang J., Zhou S., Shi Y., Qian F., Qiu S., Chen Y., Zhao D., Yang K. (2025). Current research on the design, properties and applications of tribological materials: a review. RSC Adv..

[cit7] Klekotka M., Zielińska K., Stankiewicz A., Kuciej M. (2020). Tribological and Anticorrosion Performance of Electroplated Zinc Based Nanocomposite Coatings. Coatings.

[cit8] Phogat D., Rani P., Biswas A., Balani K., Awasthi S. (2025). Architecturally Refined Cerium-Integrated Hydroxyapatite/CNT Nanocomposite Coatings: Enhanced Mechanics and Biofunction for Orthopaedic Implantation. Macromol. Biosci..

[cit9] Yu P., Zheng X., Alimi L. O., Al-Babili S., Khashab N. M. (2024). Metal–Organic Framework-Mediated Delivery of Nucleic Acid across Intact Plant Cells. ACS Appl. Mater. Interfaces.

[cit10] Altaf C. T., Colak T. O., Rostas A. M., Socaci C., Lazar M. D., Tudoran L. B., Aleinawi M. H., Misirlioglu F. B., Yildirim I. D., Erdem E., Sankir N. D., Sankir M. (2024). Zinc oxide nanoflake/reduced graphene oxide nanocomposite-based dual-acting electrodes for solar-assisted supercapacitor applications. Energy Adv..

[cit11] Weir A., Westerhoff P., Fabricius L., Hristovski K., von Goetz N. (2012). Titanium Dioxide Nanoparticles in Food and Personal Care Products. Environ. Sci. Technol..

[cit12] Praveen B. M., Venkatesha T. V., Naik Y. A., Prashantha K. (2007). Corrosion Behavior of Zn-TiO2 Composite Coating, Synthesis and Reactivity in Inorganic, Metal-Organic. Nano-Met. Chem..

[cit13] Kumar D., Khairnar Y. S., Bishwakarma H., Mardi K. B., Mandal S. K. (2025). Effective Interfacial Interaction for Enhancing the Structural and Mechanical Properties of Polydimethylsiloxane Composites Loaded With a High Concentration of Reduced Graphene Oxide-Doped Calcium Carbonate. Polym. Adv. Technol..

[cit14] Phogat D., Awasthi S. (2025). Material and technique fundamentals of nano-hydroxyapatite coatings towards biofunctionalization: a review. Biomed. Mater..

[cit15] Awasthi S., Pandey S. K., Gaur J. K., Srivastava C. (2022). Load-bearing study and interfacial interactions of hydroxyapatite composite coatings for bone tissue engineering. Mater. Chem. Front..

[cit16] Oliver W. C., Pharr G. M. (2004). Measurement of hardness and elastic modulus by instrumented indentation: Advances in understanding and refinements to methodology. J. Mater. Res..

[cit17] Hu H., Onyebueke L., Abatan A. (2010). Characterizing and Modeling Mechanical Properties of Nanocomposites-Review and Evaluation. J. Miner. Mater. Charact. Eng..

[cit18] Awasthi S., De S., Pandey S. K. (2024). Electrodeposited carbon nanostructured nickel composite coatings: A review. Heliyon.

[cit19] Khan W. S., Cao C., Zhong J., Liu Y., Iqbal M. A. (2010). Synthesis of metallic Zn microprisms, their growth mechanism and PL properties. Mater. Lett..

[cit20] Muzyka R., Drewniak S., Pustelny T., Chrubasik M., Gryglewicz G. (2018). Characterization of Graphite Oxide and Reduced Graphene Oxide Obtained from Different Graphite Precursors and Oxidized by Different Methods Using Raman Spectroscopy. Materials.

[cit21] Dervishi S., Korpa A., Gjyli S., Baydogan N., Isak N., Farruku M. (2026). Highly Efficient Ag-doped TiO_2_/graphite oxide photocatalyst for Rhodamine B degradation in wastewater treatment. J. Alloys Compd. Commun..

[cit22] Etape E. P., Foba-Tendo J., Ngolui L. J., Namondo B. V., Yollande F. C., Nguimezong M. B. N. (2018). Structural Characterization and Magnetic Properties of Undoped and Ti-Doped ZnO Nanoparticles Prepared by Modified Oxalate Route. J. Nanomater..

[cit23] Awasthi S., Pandey C. P., Balani K. (2018). Synergistic role of carbonaceous reinforcements on multi length scale tribology of electrophoretically deposited nickel-boron nitride coatings. Mater. Res. Bull..

[cit24] Awasthi S., Pandey S. K., Juyal A., Pandey C. P., Balani K. (2017). Synergistic effect of carbonaceous reinforcements on microstructural, electrochemical, magnetic and tribological properties of electrophoretically deposited nickel. J. Alloys Compd..

[cit25] Howe J. Y., Rawn C. J., Jones L. E., Ow H. (2003). Improved crystallographic data for graphite. Powder Diffr..

[cit26] Keyes B. M., Gedvilas L. M., Li X., Coutts T. J. (2005). Infrared spectroscopy of polycrystalline ZnO and ZnO:N thin films. J. Cryst. Growth.

[cit27] Castañeda L. (2020). Study of Optical Properties of Zinc Oxide Nanostructures Thin Solid Films Using Spin Coating Technique: APrecursor Organic for Electronics Devices Applications. Biomed. J. Sci. Tech. Res..

[cit28] Moon I. K., Lee J., Ruoff R. S., Lee H. (2010). Reduced graphene oxide by chemical graphitization. Nat. Commun..

[cit29] Milne A. J. B., Amirfazli A. (2012). The Cassie equation: How it is meant to be used. Adv. Colloid Interface Sci..

[cit30] Busscher H. J., van Pelt A. W. J., de Boer P., de Jong H. P., Arends J. (1984). The effect of surface roughening of polymers on measured contact angles of liquids. Colloids Surf..

[cit31] Tang K., Wang Q., Xu Z., Li Y., Zhang K., Xie L., Chang C., Qiu S., Xiao G. (2025). Micromorphology evolution and properties of strontium-zinc-phosphate chemical conversion coatings on Ti:Effect of Zn2+ and Ca2+ in the reaction solution. Surf. Interfaces.

[cit32] Tafreshi M., Allahkaram S. R., Farhangi H. (2016). Comparative study on structure, corrosion properties and tribological behavior of pure Zn and different Zn-Ni alloy coatings. Mater. Chem. Phys..

